# Piloting a programme tool to evaluate malaria case investigation and reactive case detection activities: results from 3 settings in the Asia Pacific

**DOI:** 10.1186/s12936-017-1991-9

**Published:** 2017-08-22

**Authors:** Chris Cotter, Prayuth Sudathip, Herdiana Herdiana, Yuanyuan Cao, Yaobao Liu, Alex Luo, Neil Ranasinghe, Adam Bennett, Jun Cao, Roly D. Gosling

**Affiliations:** 1 Malaria Elimination Initiative, Global Health Group, University of California, San Francisco (UCSF), 550 16th Street, 3rd floor, San Francisco, CA 94158 USA; 2 Department of Women’s and Children’s Health, International Maternal and Child Health (IMCH), Uppsala University, Uppsala, Sweden; 3 Department of Disease Control, Bureau of Vector Borne Diseases, Ministry of Public Health, Nonthaburi, Thailand; 4 Paritrana Asia Foundation, Jakarta, Indonesia; 5 United Nations Children’s Fund (UNICEF), Aceh Field Office, Banda Aceh, Indonesia; 6 Key Laboratory of National Health and Family Planning Commission on Parasitic Disease Control and Prevention, Jiangsu Provincial Key Laboratory on Parasite and Vector Control Technology, Jiangsu Institute of Parasitic Diseases, Wuxi, Jiangsu People’s Republic of China; 7 Global Health Sciences, University of California, San Francisco (UCSF), San Francisco, USA; 8 Thomson Reuters Ltd, London, UK; 9 Department of Epidemiology & Biostatistics, School of Medicine, University of California, San Francisco (UCSF), San Francisco, USA

**Keywords:** Monitoring and evaluation (M&E), Malaria, Elimination, Case investigation, Active case detection, Reactive case detection (RACD), Tool development, Programme performance, China, Indonesia, Thailand

## Abstract

**Background:**

Case investigation and reactive case detection (RACD) activities are widely-used in low transmission settings to determine the suspected origin of infection and identify and treat malaria infections nearby to the index patient household. Case investigation and RACD activities are time and resource intensive, include methodologies that vary across eliminating settings, and have no standardized metrics or tools available to monitor and evaluate them.

**Methods:**

In response to this gap, a simple programme tool was developed for monitoring and evaluating (M&E) RACD activities and piloted by national malaria programmes. During the development phase, four modules of the RACD M&E tool were created to assess and evaluate key case investigation and RACD activities and costs. A pilot phase was then carried out by programme implementers between 2013 and 2015, during which malaria surveillance teams in three different settings (China, Indonesia, Thailand) piloted the tool over a period of 3 months each. This study describes summary results of the pilots and feasibility and impact of the tool on programmes.

**Results:**

All three study areas implemented the RACD M&E tool modules, and pilot users reported the tool and evaluation process were helpful to identify gaps in RACD programme activities. In the 45 health facilities evaluated, 71.8% (97/135; min 35.3–max 100.0%) of the proper notification and reporting forms and 20.0% (27/135; min 0.0–max 100.0%) of standard operating procedures (SOPs) were available to support malaria elimination activities. The tool highlighted gaps in reporting key data indicators on the completeness for malaria case reporting (98.8%; min 93.3–max 100.0%), case investigations (65.6%; min 61.8–max 78.4%) and RACD activities (70.0%; min 64.7–max 100.0%). Evaluation of the SOPs showed that knowledge and practices of malaria personnel varied within and between study areas. Average monthly costs for conducting case investigation and RACD activities showed variation between study areas (min USD $844.80–max USD $2038.00) for the malaria personnel, commodities, services and other costs required to carry out the activities.

**Conclusion:**

The RACD M&E tool was implemented in the three pilot areas, identifying key gaps that led to impacts on programme decision making. Study findings support the need for routine M&E of malaria case reporting, case investigation and RACD activities. Scale-up of the RACD M&E tool in malaria-eliminating settings will contribute to improved programme performance to the high level that is required to reach elimination.

**Electronic supplementary material:**

The online version of this article (doi:10.1186/s12936-017-1991-9) contains supplementary material, which is available to authorized users.

## Background

Active case detection (ACD) is a World Health Organization recommended strategy aimed at detecting malaria infections at community and household level by screening individuals considered to be at high risk of malaria infection [1]. ACD strategies are designed to identify and treat malaria infections as early as possible, before they are symptomatic and reduce the reservoir of infection responsible for continued malaria transmission [2, 3].

**Fig. 1:**
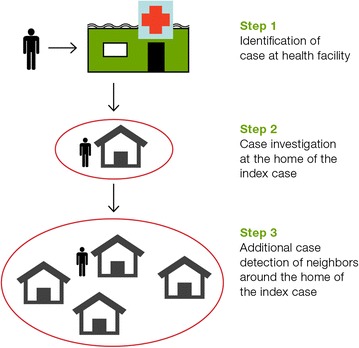
Malaria case investigation and RACD process. *Step 1* is the identification of a malaria case at the health facility. *Step 2* is a case investigation conducted by malaria personnel, typically at the home of the index case. *Step 3* is the process undertaken in a receptive area, whereby household members and neighbours of the index patient are tested for malaria

One commonly used ACD approach is reactive case detection (RACD). This surveillance and response strategy involves case investigation, whereby passively detected malaria cases (index case) are traced to their residence to determine the suspected origin of infection (local, introduced or imported infection), and ascertain if onward transmission of malaria is possible. If the area is receptive, testing of additional contacts is carried out (see Fig. 1). RACD is conducted around an index case because evidence suggests that additional malaria infections cluster in close proximity to the index household [4–6].

Despite little evidence to support the impact of RACD on transmission, RACD has been widely adopted with methodologies varying across eliminating settings [4, 7–10]. RACD is time and resource intensive, and, in the low endemic areas where RACD is implemented using diagnostics with poor sensitivity for detecting low parasite densities, yields few additional cases [11–13]. Further, no standardized metrics or tools exist to monitor and evaluate RACD activities [7, 14]. In response to the lack of monitoring and evaluation (M&E) of index case investigation and RACD activities, a simple programme tool was developed to support these activities by national malaria programmes. In this paper the design and implementation of the RACD M&E programme tool is described, and the results of three pilots carried out in low-endemic settings in the Asia Pacific.

## Methods

This project involved two phases: (1) a tool development phase, during which the components of the RACD M&E tool were developed; and, (2) a pilot phase, during which malaria surveillance teams across three different programme settings piloted the tool.

### The RACD M&E tool

The RACD M&E tool is a mixed-methods process evaluation to assess malaria case reporting, case investigation, and RACD activities through the collection of quantitative and qualitative data. The RACD M&E tool was developed between April and June 2013. The tool is a set of documents and templates based on a Microsoft Excel and Word platform for ease of use, broad familiarity and wide availability of software.

The RACD M&E tool is designed to guide national malaria programmes in assessing the performance of staff conducting RACD-related activities and identifying what activities can be improved to strengthen surveillance. The RACD M&E tool assesses key components of the RACD process starting when the index patient is confirmed at the health facility through the index case follow-up and investigation, and the malaria personnel response when the index case household and surrounding community members of the patient are screened. The evaluation is designed to be led by national or provincial programme personnel, typically surveillance officers, in coordination with district and health facility-based staff in the target evaluation areas. The RACD M&E tool is divided into four components (referred to as modules—see Table 1) based on data collection methods and timing and are described in detail below. Each module automatically calculates outputs of summary statistics and proportions tailored to the data collected in that module.

**Table 1:** Overview of RACD M&E tool modules

Module 1: reviewing key documents	Objective: to review the key documents and personnel involved in the RACD process and determine the availability and use of reporting forms at the health facility level, including:
	Organizational diagrams
	Standard operating procedures (SOPs)
	Malaria case notification, case investigation and RACD reporting forms
	Activity and reporting flow diagrams
Module 2: assessing key malaria indicators	Objective: to compare and evaluate the accuracy of malaria case reporting, case investigations and RACD activities using indicators of:
	Completeness and timeliness of health facility reporting
	Case investigation completeness and timeliness
	RACD completeness, timeliness, screening^a^ coverage and positives identified
Module 3: evaluating standard operating procedures (SOPs)	Objective: to evaluate the baseline knowledge and practices of programme staff on SOPs and understand existing gaps and challenges in conducting case investigation and RACD activities, including:
	Minimum screening radius around an index case
	Household and community individuals to target and screen
	Practices for screening during follow-up visits
	Challenges to conducting case investigations and RACD
Module 4: estimating the costs	Objective: to estimate the costs of conducting case investigation and RACD activities at district and provincial levels, including the main cost drivers for:
	Malaria surveillance personnel (paid and volunteer)
	Commodities for malaria activities
	Services and other costs

*RACD* reactive case detection, *SOP* standard operating procedures

^a^Diagnostic used according to local standard guidelines

### Tool modules

#### Module 1. Reviewing key documents

A review of key documents for personnel involved in the RACD process, standard operating procedures (SOPs), case notification forms, organizational diagrams, and activity and reporting flow diagrams are reviewed to determine how formalized the RACD process is. The key documents are reviewed to determine if they exist, and are used by appropriate staff that report malaria cases and participate in RACD activities. Module 1 uses an Excel template to guide the evaluator through the review of key documents. Automated outputs of the reviewing key documents module show summary proportions for each health facility component being inventoried (Additional file 1).

#### Module 2. Assessing key malaria indicators

The minimum essential indicators on index case notification/reporting, case investigation and RACD timeliness and completeness at health facility and district levels are collected and assessed. District-level personnel visit all health facilities (or a selected sample depending on programme need, such as the highest burden or more remote facilities) to compare patient log books with the malaria surveillance database to identify gaps in completeness and timeliness of reporting cases. Case investigation and RACD indicators are aggregated to the district level and evaluated for completeness, timeliness, total individuals screened and additional positives identified during RACD. Raw data are manually entered into the Excel tool template by programme personnel. The Excel spreadsheet automatically calculates the key data indicators and produces them in a report format (Additional file 2).

#### Module 3. Evaluating standard operating procedures

An open- and closed-ended questionnaire-based module using Word is completed by all health facility and malaria surveillance personnel conducting case investigation and RACD activities (Additional file 3). Module 3 evaluates the baseline knowledge and practices of programme staff on the SOPs they are supposed to follow. The assessment identifies any existing gaps and challenges in conducting case investigation and RACD activities. Questionnaire responses are collated and entered into the Excel template. Outputs of this module show summary proportions of the malaria personnel responses and can be filtered by evaluation area (Additional file 4).

#### Module 4. Estimating the costs

To understand the costs associated with conducting case investigation and RACD activities, the RACD M&E tool includes a module to calculate monthly and annual expenditures. Costs for case investigation and RACD activities are collected from district and provincial sources such as budgets, invoices, salary and field logs, fuel receipts, supply orders and other documentation. Programme personnel manually input the costing data into the Excel template. Main cost categories include personnel, commodities, services, and other costs. All costs are entered in local currency. The Excel spreadsheet automatically calculates summary figures and tables and produces them in a report format (Additional file 5).

RACD M&E tool implementation should be led by a lead programme surveillance officer at the national or provincial level. The RACD M&E tool can be found online [15].

### Pilot phase

#### Study areas

Pilot areas were selected by agreement with national or provincial malaria programmes through the surveillance and response working group (SRWG) of the Asia pacific malaria elimination network (APMEN) [16]. Three pilot sites were selected: (1) Aceh Province, Indonesia; (2) Jiangsu Province, China; and, (3) Ranong Province, Thailand. Sites were selected based on whether national malaria control programmes were currently conducting case investigation and RACD activities at the time, and had an interest in conducting the pilot. Malaria transmission levels, reporting structures, and scope of evaluation varied among pilot areas and are summarized in Table 2. Each pilot site implemented the RACD M&E tool for 3 months, during 2013–2015, using the most recent 12 months of malaria programme data (Aceh Province evaluated 3 months of data). This retrospective evaluation allowed pilot sites to evaluate their activities over a full year, covering all transmission seasons.

**Table 2:** Summary of pilot study areas

Location	Pilot implementation period	Data collection period	Phase	Pilot scale	Health facility catchment area population	Total number of facilities	Number of staff interviewed
Aceh, Indonesia	June–September 2013	June–September 2013	Elimination	Five subdistricts^a^	1,343,849	34/34	34
Jiangsu, China	June–August 2013	January–December 2012	Prevention of reintroduction	Three counties^b^	10,149,000	6/6	10
Ranong, Thailand	January–March 2015	January–December 2014	Elimination	Five districts^c^	177,089	10/10	15

^a^ *Aceh* Subdistricts: Aceh Besar, Aceh Timur, Banda Aceh, Bireun, Sabang

^b^ *China* Counties: Baoying, Gulou (Nanjing City), Haimen

^c^ *Thailand* Districts: Kapoe, Kraburi, Laun, Meaung, Suksamran

### Training for the pilot evaluation

Prior to implementation, brief in-person training sessions were conducted with provincial and national-level malaria surveillance officers to familiarize them with the purpose and objectives of the RACD M&E tool. Tool modules and templates, objectives of the pilots and questions about its use were reviewed and discussed. Minimal adaptation of the modules, including data indicators and questionnaires was permitted by study areas prior to implementation in order to maintain standardization across the pilots. Questionnaires and data collection tools were translated into the local language, as necessary, by the pilot site implementers so that district- and health facility-level personnel could review and input data.

### Study procedures

National—(Thailand) and provincial-level (China, Indonesia) staff led the field data collection activities. Health facility data were recorded by manually counting patient registers and entering totals into the tool template. Case investigation and RACD indicator data were collected from the malaria database by the lead pilot implementers for each site and entered manually into the Excel template. Costing data were collected in coordination with provincial- and district-level programme support, depending on where the expenditure records were kept within country. Questionnaires were administered in-person and responses entered manually into the tool template. Data cleaning and quality control was monitored by the lead pilot implementers at each site and reviewed by RACD M&E tool developers prior to data analysis.

### Assessment of RACD M&E tool

A feedback workshop was held with programme implementers for China and Indonesia pilots in Jiangsu, China in October 2013 to review the preliminary results of the pilots and obtain feedback. The third pilot area (Thailand) was visited in March 2015 to review pilot results and obtain feedback. In addition to analysis of the RACD M&E tool data collected, pilot implementers reported on the tool’s ability to identify gaps and challenges in RACD programme activities, as well as qualitative assessments on the difficulties experienced during implementation, general suggestions to improve each tool module and data collection sheets, and the overall benefit of having an evaluation tool for malaria case reporting, case investigation, and RACD activities. In addition to the workshop, qualitative feedback was gathered from programmes individually via face-to-face visits following the pilot phase, and through informal discussions since those visits, on the impact the tool had on programme decision making. During the qualitative feedback discussion, pilot site implementers were asked about whether they planned additional roll-out of the tool and the scale to which it will be implemented, what monitoring activities and/or trainings were put in place after the pilots, whether the indicators included were the most appropriate to include in the tool, and other general improvements that were made to programme activities based on the results of the pilots.

## Results

All three study areas successfully implemented the four tool modules. Summary results for each of the four modules are shown below.

### Tool modules

#### Module 1. Reviewing key documents

The reviewing key documents module showed that not all health facilities in the study areas had the proper notification forms, SOPs or documentation to support malaria implementation activities. In Aceh, Indonesia 70.6% (24/34) of health facilities examined had a case notification form available. Eight per cent (3/34) of health facilities examined in Aceh had SOPs or instructions on the reporting processes for case notification, investigation or RACD; Ranong, Thailand had zero (0/5) and Jiangsu, China reported 100% (6/6). Nearly all study areas had organizational diagrams of the malaria personnel involved in case investigation and RACD activities [88.2% (30/34) in Aceh; 100.0% (6/6) in Jiangsu; 100.0% (5/5) in Ranong]. Table 3 summarizes the results of module 1.

**Table 3:** Reviewing key documents module results

Inventory list for each health facility	Aceh		Jiangsu		Ranong		Total	
	(n = 34)		(n = 6)		(n = 5)		(n = 45)	
	Available	%	Available	%	Available	%	Available	%
Is there a diagram of the malaria personnel organizational structure?	30	88.2	6	100.0	5	100.0	41	91.1
Does it include both paid and volunteer personnel?	3	8.8	6	100.0	5	100.0	14	31.1
Health facility case notification form available?	24	70.6	6	100.0	5	100.0	35	77.8
Index case investigation form available?	28	82.4	6	100.0	5	100.0	39	86.7
RACD form available?	12	35.3	6	100.0	5	100.0	23	51.1
*Reporting forms availability subtotal*	*64/102*	*62.7*	*18/18*	*100.00*	*15/15*	*100.0*	*97/135*	*71.8*
SOPs for health facility diagnosis and notification?	3	8.8	6	100.0	0	0.0	9	20.0
SOPs for index case investigation?	3	8.8	6	100.0	0	0.0	9	20.0
SOPs for RACD?	3	8.8	6	100.0	0	0.0	9	20.0
*SOP availability subtotal*	*9/102*	*8.8*	*6/6*	*100.0*	*0/5*	*0.0*	*27/135*	*20.0*
Diagram of process for health facility diagnosis and reporting?	3	8.8	6	100.0	0	0.0	9	20.0
Diagram of process for case investigation?	3	8.8	6	100.0	0	0.0	9	20.0
Diagram of process for RACD?	3	8.8	6	100.0	0	0.0	9	20.0
*Diagram availability subtotal*	*9/102*	*8.8*	*6/6*	*100.0*	*0/5*	*0*	*27/135*	*20.0*

*SOPs* standard operating procedures, *RACD* reactive case detection

#### Module 2. Assessing key malaria indicators

Key malaria indicator results for each pilot area showed that gaps exist in reporting completeness and timeliness. Of note in Table 4, Indonesia identified gaps in malaria case reporting completeness (93.3%, 112/120) between the health facility patient registers and the district-level malaria database. Indonesia and Thailand study areas identified gaps in health facility reporting timeliness (88.3%, 106/120; 50.8%, 259/510, respectively), while China reported 100.0% (42/42) for completion and timeliness. Indonesia and Thailand investigated 78.4% (87/111) and 61.8% (465/752) of index cases, and of those cases that were investigated, 98.3% (57/58) and 64.7% (271/419) had RACD events completed (out of total RACD events that should occur), respectively. Additional summary results can be found in Table 4.

**Table 4:** Key indicator results from pilot study areas

Indicators	Aceh		Jiangsu		Ranong		Total	
	No.	%	No.	%	No.	%	No.	%
Malaria cases reported to the database from health facilities	112/120	93.3	42/42	100	510/510^e^	100.0	664/672	98.8
Malaria cases reported to the database within a specific amount of time^a^	106/120	88.3	42/42	100	259/510^e^	50.8	407/672	60.5
Malaria cases reported to the database that were investigated	87/111	78.4	42/42	100	465/752	61.8	594/905	65.6
Malaria cases reported to the database that were investigated within a specified amount of time^b^	79/87	90.8	42/42	100	394/465	84.7	515/594	86.7
RACD events that occurred (out of total RACD events that should occur^d^)	57/58	98.3	19/19	100	271/419	64.7	347/496	70.0
RACD events that occurred within a specified amount of time^c^	47/58	81.0	19/19	100	229/271	84.5	295/348	84.7
Total population screened during RACD events	931	–	n/a	–	18,505	–	19,436	–
Positive malaria cases identified through RACD	3	–	0	–	26	–	29	–

*n/a* data not available, *RACD* reactive case detection

^a^Number of days varies by study area: China (1); Thailand (3); Indonesia (30). Aceh Province health facilities report malaria cases to district on a monthly basis (except for Sabang health facilities which reports malaria cases within 1 day)

^b^Number of days varies by study area: China (3); Thailand (3); Indonesia (30)

^c^Number of days: China (7); Thailand (7); Indonesia (7)

^d^Number of RACD events required is based on local stratification criteria determining receptive areas

^e^Sample of total cases in province

#### Module 3. Evaluating standard operating procedures

Questionnaires administered to malaria personnel that conduct case investigation and RACD activities showed that practices varied widely. Differences were identified when asked about the minimum geographic screening radius for RACD: respondents in China did not have a clear majority response with 40% (4/10) stating 50–100 m radius, Indonesia stating 100 m (44%, 15/34) and Thailand stating 1 or 2 km (18% each, 2/11). When asked about which individuals should be screened during RACD, a majority of respondents across all 3 study sites stated that “all asymptomatic and febrile” individuals should be screened: China 70% (7/10), Indonesia 50% (17/34), and Thailand 73% (8/11). Table 5 highlights key SOP evaluation findings (Additional file 6).

**Table 5:** Sample of key findings from evaluating standard operating procedures in pilot areas

Aceh			Jiangsu			Ranong		
Responses	n	%	Responses	n	%	Responses	n	%
1. Question: what is the minimum geographic radius to screen around an index case household during RACD?								
100 m	15	44	50–100 m	4	40	***1 km***	***2***	***18***
200 m	1	3	150–200 m	2	20	***2 km***	***2***	***18***
***500*** ***m***	***13***	***38***	***1*** ***km***	***1***	***10***	3 km	2	18
No radius	2	6	No radius	2	20	5 km	1	10
No response	3	9	No response	1	10	No radius	4	36
Total	**34**	**100**	Total	**10**	**100**	Total	**11** ^a^	**100**

Responses	Aceh		Jiangsu		Ranong	
	n	%	n	%	n	%
2. Question: Which individuals should you screen when conducting RACD around an index case household?						
Febrile only	14	41	2	20	1	9
***All (asymptomatic and febrile)***	***17***	***50***	***7***	***70***	***8***	***73***
We do not screen household members of a positive case	3	9	1	10	2	18
Total	**34**	100	**10**	100	**11** ^a^	100
3. Question: what triggers screening in the community?						
Local cases only	5	15	0	0	2	18
***Local and imported cases***	***26***	***76***	***10***	***100***	***7***	***64***
Imported cases only	0	0	0	0	0	0
When local cases reach a minimum threshold	0	0	0	0	0	0
Neighbors of index case never screened	3	9	–	–	–	–
Total	**34**	100	**10**	100	**9** ^a, b^	100
4. Question: how often do you screen neighbors around index case household?						
***We always screen neighbors***	***13***	***38***	***6***	***60***	***11***	***73***
Sometimes screen neighbors	18	53	3	30	4	27
Never screen neighbors	3	9	1	10	0	0
Total	**34**	100	**10**	100	**15**	100

^a^Four individuals do not conduct screening in the community

^b^Two individuals did not respond

Correct responses highlighted in bold and bolditalics

#### Module 4. Estimating the costs

Costs were collected for pilot areas on case investigation and RACD-related expenses and separated into two main categories: (1) personnel, and (2) commodities, services and other. Cost totals showed variation between study areas for both categories ranging from $3469 (Indonesia) to $10,486 (Thailand) for total personnel and $257 (Indonesia) to $13,969 (Thailand) for commodities, services and other costs. For comparison, total average monthly costs were estimated across the three pilot sites for China ($844), Thailand ($2038) and Indonesia ($3727) with different numbers of malaria programme personnel included. Table 6 summarizes the main costing estimate results for case investigation and RACD activities from the pilot areas.

**Table 6:** Costing summary results from pilot study areas

Study area	Data collection period	Personnel^a^		Commodities, services and other		Total cost		Average monthly cost^b^	
		All malaria activities	CI/RACD only	All other malaria activities	CI/RACD only	All malaria activities	CI/RACD only	All malaria activities	CI/RACD only
Aceh	September 2013 (1 month)	n/a	$ 3469.56	n/a	$ 257.55	n/a	$ 3727.11	n/a	$ 3727.11
Jiangsu	January–December 2012 (12 months)	$ 9101.13	$ 4550.56	$ 5513.89	$ 5587.08	$ 20,202.10	$ 10,137.64	$ 1683.50	$ 844.80
Ranong	January–December 2014 (12 months)	n/a	$ 10,486.61	n/a	$ 13,969.43	n/a	$ 24,456.04	n/a	$ 2038.00

All costs converted from local currency to US dollars ($), adjusted for inflation, and shown in 2016 USD$

*n/a* data costs not available, *CI* case investigation, *RACD* reactive case detection

^a^Number of personnel included for each study site: Aceh (29), Jiangsu (3), Ranong (28)

^b^Average monthly costs equals total cost divided by number of months in the data collection period

### Assessment of RACD M&E tool

All three study areas that implemented the RACD M&E tool agreed that, based on the findings, gaps and challenges identified during the pilots, the tool and evaluation process was helpful to identify gaps in RACD programme activities, and should be further rolled out. Pilot users reported several challenges during the implementation of the RACD M&E tool and made suggestions to improve tool functionality for future programme implementation.

### Suggested changes to tool

Challenges with implementation of the modules were experienced by each pilot area. One challenge noted that despite brief instructions included on the tool pages, users were unsure of how to complete each of the module sections. Furthermore, users found it difficult to know exactly which data to include in the Excel template (particularly in the costing and data indicators modules). Pilot users suggested that in addition to the brief instructions on data entry pages, having user manuals that include more detail on the module sections and data requirements will support data entry and RACD M&E tool use.

All three study sites needed to translate data collection template pages because the RACD M&E tool templates were created in English. Before going to the field or sending template sheets to be filled in by district-level staff, pilot implementers found that translating each template page was additional work. Users suggested having a central translation worksheet within each module that made translating the modules easier to save time and allow programmes the flexibility to modify the translations to fit the local context. The central translation worksheet would be translated at a central level prior to distribution more broadly throughout the country. Pre-set drop-down lists, in local language, were also suggested to standardize the template when marking the different province and district to be evaluated.

Pilot users highlighted difficulty in completing the data indicators template because it was separated into three sheets: health facility, case investigation and RACD. Users were required to switch between data entry sheets making the data entry process longer and more confusing. Users suggested a template design that included two data entry sheets more appropriate for the typical local context and reporting structure: one for health facility-level data and one for the district-level to capture all case investigation and RACD data. Malaria programmes stated they want the option to monitor a sample or all health facilities depending on the size of the evaluation area and their evaluation needs. Users maintained, however, that case investigation and RACD indicators should continue to be aggregated to the district level and be included in a single data collection sheet. Furthermore, Excel-based macros were suggested as a way to increase the ability for national- and/or provincial-level programme staff to be able to aggregate district-level data. Programmes further stated that the indicator for total numbers of individuals screened during RACD is insufficient because they need to know what proportion of the total population is screened during RACD in an area (screening coverage). Pilot users suggested it would be more helpful to include the total number of all individuals living in the screening area to better understand how many individuals are being missed during RACD screenings. See Table 7 for more suggested changes to the RACD M&E tool modules and templates.

**Table 7:** Suggested changes to the RACD M&E tool from 3 pilot settings

	Suggestions by programme implementers
RACD M&E tool overall	Create user manuals for district- and national-level programme staff for each module to provide more detail on the inputs required for data collection
	Enable translations for each template sheet to increase use of tool
	Create automatic data analysis pages in each module for district-level monitoring
	Develop macros in Excel to allow provincial- and national-level users to compile results across districts
Module 1: reviewing key documents	Create preset drop-downs with yes/no responses for inventory checklist items
Module 2: assessing key malaria indicators	Reduce the number of data entry sheets from 3 (health facility reporting, case investigation and RACD) to 2 (health facility data, district level data) by combining case investigation and RACD into a single data collection sheet
	Simplify the district-level indicators sheet for case investigation and RACD by including all the indicators on a single data entry sheet, separate from the review pages
	Create option for preset drop-down lists to make data entry quicker, and to maintain consistency in the spelling of district and province names
	Add indicator for RACD screening coverage
Module 3: evaluating standard operating procedures	Develop questionnaire in open source platform or improve data analysis in current Excel template to make it easier
	Allow programme users to modify the questionnaire module to match programme activities and needs
Module 4: estimating the costs	Reduce the number of data entry sheets from 3 (Personnel, Commodities and Services and Other) to 2 (Personnel and Commodities, Services and Other) into a single data collection sheet. Include a drop-down list to identify the expense

### Impact on programme decision making

Pilot implementers noted during the feedback assessments that the RACD M&E tool findings had an impact on programme activities. In addition to broadening the use of the RACD M&E tool to other districts and provinces in pilot countries, all three programmes identified that additional training sessions were needed for programme surveillance staff. These included refresher trainings on using standardized reporting forms and ensuring that case investigations and RACD response activities are being carried out according to SOPs.

Users noted that based on the RACD M&E tool results, improvements to RACD SOPs were made and adapted to the local situation where necessary. For the programmes that were not already collecting data on completeness and timeliness indicators presented in this tool, users stated that they have since included them in their malaria reporting systems. All three pilot areas noted that the pilot findings will support discussions with programme managers and policy makers on the need to incorporate these routine M&E activities into their surveillance guidelines. Furthermore, these findings support surveillance managers when they ask for the required budget allocations to include additional surveillance personnel to conduct greater monitoring activities. Table 8 highlights the reported impacts that the RACD M&E tool has had on the pilot user’s programmes.

**Table 8:** Impact of the RACD M&E tool findings on programme decision making in 3 pilot settings

Study site	RACD M&E tool impact on programme decision making
Aceh, Indonesia	Broadened use of RACD M&E tool to other districts in Aceh Province and other provinces within Indonesia
	Recommend the integration of the M&E tool into the current national malaria case reporting system (referred to as e-SISMAL)
	Conducted refresher trainings with malaria officers and microscopists at district level on the knowledge and information at primary health centers through routine meetings
	Recommended to provincial health office on the use of standardized case investigation and RACD forms for entire province, and need to undertake a notification form from the ministry of health that is under the responsibility of the surveillance unit
	Advocated to district and provincial health offices to allocate more budget for supervision and field monitoring
	Set up random monitoring of malaria program implementation in Aceh for quality assurance of activities and reporting
	Recommend the development of a tool for M&E in malaria diagnosis QA system to be created and tested in Sabang and Aceh Besar Districts before scaling up nationally
Jiangsu, China	Carried out additional evaluations in Jiangsu and Yunnan Province in China
	Improved China’s 1-3-7 SOPs in Jiangsu, making the SOPs more suitable for the local context
	Added indicators of completion rates for the China’s national 1-3-7 reporting framework to the routine diseases surveillance information system (CRDSIS)
	Jiangsu Institute of Parasitic diseases (JIPD) malaria division carried out additional trainings on malaria reporting system management, epidemiology investigation and foci disposals for basic level CDCs staff in Jiangsu province
Ranong, Thailand	Adopted routine monitoring and evaluation activities into national surveillance guidelines
	Incorporated into online database for all reporting facilities standardizing indicators for routine reporting
	Conducted refresher trainings on case investigation and RACD because gaps identified differences in how activities were conducted
	Recommend to conduct a full national evaluation using the M&E tool
	Recommended including a rapid reporting system because gaps were identified from remote clinic areas (malaria post, border malaria post) and malaria clinics without computers/internet have reports that are not timely/complete
	Recommend integration of the case investigations into the primary health system to not miss case investigations and to be more timely/complete

## Discussion

Piloting of a simple RACD M&E tool in three settings in the Asia Pacific region identified programme gaps and resulted in measures being put in place to improve performance in malaria case reporting, case investigation and RACD. The tool allowed national malaria control programmes (NMCPs) to discover that malaria case reporting, case investigation and RACD activities have gaps in completeness and timeliness, as well as knowledge and practice differences in surveillance personnel. Impact of the RACD M&E tool findings on programme activities include enhanced training sessions on case investigation and RACD, adopting new indicators for enhancing surveillance, improving SOPs on RACD practices, and providing evidence that gaps exist in the programme performance. To our knowledge, this is the first comprehensive M&E tool developed for malaria programmes conducting case investigation and RACD.

Study findings support the need for routine M&E of malaria case investigations and RACD activities as a necessary component of malaria elimination programmes [17]. With the quality of a surveillance system being only as good as its implementation, the RACD M&E tool study findings demonstrate that the low performance of some of the pilot sites may likely be a barrier to them achieving malaria elimination [18]. A number of factors can reduce the overall effectiveness of a malaria programme to follow up all index cases and screen household members and neighbours. One factor in determining the effectiveness of malaria case investigation and RACD depends largely on how well the programme is carrying out the reporting and follow-up activities. If poorly conducted, index cases will not be followed up, potentially allowing for continued malaria transmission around the index case household. This analysis shows that not all index patients who were confirmed malaria positive at the health facility were reported, or had a case investigation or RACD follow-up event conducted (Table 4).

The RACD M&E tool identified that case notification forms and SOPs were not uniformly distributed or used in all health facilities reporting malaria cases and conducting follow-up. Programmes reported that they conduct annual refresher trainings on surveillance; however, these findings show that not all pilot areas had the tools necessary to report malaria cases or the understanding of how and when to report cases and conduct follow-up. A total of 20% (9/45) of the pilot areas had SOPs or diagrams of the reporting process, potentially limiting their understanding of the activities they should be completing. Having these tools available and enforcing their use in all reporting facilities, much in the way China has done with mandatory malaria case reporting laws [8], will support uptake and improved reporting practices.

Previous evaluations of malaria case data have been conducted, yet they typically evaluate only the completeness and timeliness of malaria data entered into the surveillance database [19–21]. However, this alone does not provide an accurate picture of all the gaps in the case investigation and RACD process. This study shows that when health facility patient registers are examined, even when a sample of all health facilities reporting malaria cases is assessed, discrepancies are identified (Table 4). Regular monitoring of health facility malaria case registers by visiting health facilities or calling malaria personnel based there will support greater accuracy in reporting of cases to ensure all passively-detected index cases receive follow-up investigations.

Evidence has shown that the timeliness of reporting malaria cases and conducting the follow-up response is important to identify additional cases [4]. Results in pilot areas highlight the reported follow-up times ranged from 81 to 100% for the time-bound parameters set by each study area (Table 4). Similarly, gaps in index case investigation and RACD follow-up completeness and timeliness show that index cases were not followed up and RACD activities not conducted, potentially leading to continued transmission in receptive areas. These gaps may be due to difficult terrain to reach index patient communities or a lack of health facility workers to conduct malaria activities, especially if the staff are managing other diseases, as they are in the Aceh and Jiangsu Provinces. This leads to delays in reporting and RACD follow-up.

The coverage of individuals screening during RACD is important when conducting follow-up around an index case. A number of factors should be considered such as the time of day the screening occur, screening refusals, status of individuals being screened (febrile or afebrile) and total screening coverage of the population that should be screened. Tracking the coverage achieved during RACD is equally important to ensure the right individuals are reached and screened, and the reason why a particular individual might refuse screening. Pilot programmes suggested including a coverage indicator instead of only collecting the total numbers screened during RACD to know what proportion of the population has been screened. One challenge is that many programmes in low transmission settings lack household-level listings of all the individuals residing in an area. Total village population numbers may exist, but that does not provide an accurate coverage indicator of who should be screened around an index case. Programmes should consider including an indicator on total population to be screened during RACD, update routine data collection forms to include the total number of individuals living in each household they visit, and to store that information in a central location for future use and updating as necessary.

Study findings highlight the need for SOPs on malaria case reporting, investigation and RACD, given that they do not exist at many of the health facilities examined in this study. In China, where SOPs do exist, there is a need to adapt the activities described in the SOPs to the local programme setting. Once SOPs are established and adapted, additional training should be conducted for surveillance personnel to standardize their daily activities. It has been documented that case investigation and RACD practices are not standardized [7]. Study findings demonstrate a lack of knowledge on case investigation and RACD policies and different practices amongst surveillance staff in all 3 study pilot areas. A lack of knowledge of the case investigation and RACD guidelines may likely be a result of not having SOPs available, and include wide-ranging practices on different screening radius, screening populations, and RACD triggers for screening in the community (Table 5). SOPs specific to a particular setting with detailed instructions and diagrams for each step of the process and highlighting who is responsible for what part of the process will promote standardization across a programme as well as accountability and transparency in case reporting, investigation and RACD follow-up.

Pilot users collected personnel, commodities, services, and other costs specific to case investigation and RACD activities, highlighting great variance in the cost drivers between countries. The difference is likely due to programme structure and level of integration of malaria-related activities into the broader healthcare system. For example, of the two pilot programmes that collected 12 months of cost data, Ranong Province, Thailand, had higher annual cost during the pilot period. Thailand has a vertical malaria programme structure with malaria clinics and border malaria posts staffed by malaria-specific personnel and commodities earmarked for malaria activities. Compared with Thailand, Jiangsu, China, had lower annual costs largely due to a shorter malaria transmission season, as well as having an integrated health system structure with malaria personnel and activities based at the health facility level. Programme integration of vector-borne disease-specific staff (including malaria, filariasis and dengue) can be a way to save costs and share resources across similar vector borne diseases without sacrificing key vector control activities. Understanding the costs and cost-drivers for case investigation and RACD-specific activities can provide district-, provincial- and national-level managers with the information they need to plan, budget and advocate for the resources necessary to maintain a strong active surveillance programme.

All three programmes thought the RACD M&E tool was helpful to identify programme gaps in RACD activities, and each have plans to expand tool implementation. Modifications have been made to the RACD M&E tool to allow for adaptation to different programme settings. Included in the module templates will be standard translations and set-up pages that can allow programmes to make data entry easier and quicker to use which will promote increased uptake of the tool.

Programmes can use the evaluation tool retrospectively in districts already conducting RACD, as was done in the pilots, or prospectively for monitoring. Based on feedback from programmes, user manuals were developed that are specific to each module and targeted for the intended user, either district-level or provincial-/national-level personnel.

The key malaria indicators module can be implemented with several years of data for health facility reporting, case investigation and RACD to identify any patterns or differences in reporting completeness and timeliness. For programmes that have recently begun conducting these activities, or would like to begin, the RACD M&E tool can be used as a guide, with templates available for data entry, on the essential steps to monitor and evaluate in case investigation and RACD activities. Once compiled, these data can be useful in exploring trends over time and may be a stepping stone towards more comprehensive, web-based reporting and monitoring systems such as the District Health Information System 2 (DHIS2) [22]. The RACD M&E tool can be downloaded by malaria programmes that see a need for its use [15].

Several limitations to the RACD M&E tool pilots should be noted, including interviewer bias when obtaining the informal face-to-face feedback and self-report bias with use of the questionnaires. The questionnaire included general questions about case investigation and RACD activities which allowed for the questionnaires to be tested across multiple settings and see what worked. However, if the questionnaire was tailored to a particular country context, more specific responses could have been elicited by the programmes. There was no independent observation of activities to confirm the field practices, although this is something malaria programmes can undertake as a way to triangulate the results from the indicator data and qualitative responses.

Adoption of the RACD M&E tool in all low transmission settings that are conducting or planning to begin case investigation and RACD activities should be considered to promote greater routine monitoring and evaluation of those activities. Similar tools can be developed for other aspects of the surveillance and response process to strengthen and expand current M&E frameworks for malaria elimination programme operations including, foci investigation and response activities, private sector reporting, entomological assessments, and pro-active case detection in high-risk populations, among others.

## Conclusions

Malaria programmes conducting case investigation and RACD should consider integrating the RACD M&E tool modules and process into their routine programme activities with the goal to achieve maximum performance, an essential component to reaching malaria elimination. Programme managers, surveillance personnel and policy makers can use the RACD M&E tool findings to improve the quality of programme activities as well as decision making for greater programmatic success. Global and regional policy makers should adopt the proposed indicators outlined here for case reporting, case investigation and RACD to standardize and improve performance in surveillance activities for malaria elimination.

## Additional files

**Additional file 1.** Module 1 Reviewing key documents Excel template.

**Additional file 2.** Module 2 Assessing key malaria indicators Excel template.

**Additional file 3.** Case investigation and reactive case detection evaluation - key questions.

**Additional file 4.** Module 3 Evaluating standard operating procedures Excel template.

**Additional file 5.** Module 4 Estimating the costs Excel template.

**Additional file 6.** Module 3 results—questionnaire responses for the 3 pilot settings.

## Abbreviations

RACD: reactive case detection
CI: case investigation
M&E: monitoring and evaluation
SOP: standard operating procedures
