# Supplementary material for: Piloting a programme tool to evaluate malaria case investigation and reactive case detection activities: results from 3 settings in the Asia Pacific
Source: Malar J. 2017 Aug 22;16:347. doi: 10.1186/s12936-017-1991-9 (PMC5568298; doi:10.1186/s12936-017-1991-9)
Supplement: Supplementary file 3 — Additional file 3. Case investigation and reactive case detection evaluation - key questions. [file 12936_2017_1991_MOESM3_ESM.docx]

**Additional file 3: Case Investigation and Reactive Case Detection Evaluation – Key Questions**

**I. Case investigation**

***CLOSE-ENDED***

1. What is the policy for conducting a case investigation? (choose one)
   1. All indigenous and imported cases
   2. Indigenous cases only
   3. Imported cases only
   4. Other – Please specify:
2. When doing a case investigation, which of the following best describes how you make an appointment with that index case? (choose one)
   1. Telephone the index case
   2. No communication - go to the index case residence to see if he or she is home
   3. Other – Please specify:
3. What do you do if the index case is not home when you visit? (choose all that apply)
   1. Visit a second time: later that day or on a subsequent day
   2. Telephone to schedule an appointment
   3. We mark the case as imported
   4. We mark the case as “not found”
   5. We do not re-visit the index case
   6. Inform volunteers to make appointment with the case
   7. Other – Please specify:
4. What time of day do you normally investigate cases? (choose all that apply)
   1. Right after case detected at health facility
   2. Before 8am
   3. Between 8am-12noon
   4. Between 12noon-5pm
   5. After 5pm
   6. Weekends
   7. Whenever the team is available
5. What information do you use to determine if an index case is imported or local?
   1. Case has traveled to another endemic area (choose all that apply)
      1. Village
      2. District
      3. Province/State
      4. Region
      5. Country
      6. Any of the above
      7. Other – Please specify:
6. If all cases are not investigated, what are the main reasons these cases are not investigated?

(Circle all that apply)

- 1. It is an imported case
  2. It is outside of my district
  3. The person could not be found
  4. Not enough staff/resources
  5. Daily cross-border case
  6. Not applicable – we investigate every case
  7. Other – Please specify:

***OPEN-ENDED***

1. What are some of the challenges in conducting case investigation?

**II. Reactive Case Detection**

***CLOSE-ENDED***

1. Do you screen household members of the index case?
   1. Always
   2. Sometimes – Please explain:
   3. Never

1. When screening household members do you screen:
   1. Febrile cases only
   2. All household members (asymptomatics and febrile cases)
   3. We do not screen household members of a positive case
2. What do you do if someone from the household of the index case is not home and you cannot screen them? (Check all that apply)
   1. Visit the household later that day or on a subsequent day
   2. Schedule an appointment with the household members to return
   3. We do not return
   4. Other – Please specify:
3. How often do you screen neighbors of the index case in the community?
   1. Always
   2. Sometimes
   3. Never
4. What triggers screening in the community?
   1. Local cases only
   2. Local and imported cases
   3. Imported cases only
   4. When local cases reach a minimum threshold
      1. Minimum threshold of cases:
5. Do you screen a minimum number of households around a positive index case? Yes/No
   1. Number of households screened:
6. Do you screen a minimum number of people around a positive index case? Yes/No
   1. Number of people screened:
7. Do you screen within a minimum geographic radius around a positive index case? Yes/No
   1. Number of meters radius screened:
8. What time of day do you normally conduct screening in the community?

(Circle all that apply)

- 1. Right after case is detected at the health facility
  2. Before 8am
  3. Between 8am-12noon
  4. Between 12noon-5pm
  5. After 5pm
  6. Weekdays
  7. Weekends
  8. Whenever the team is available

1. If someone is missing at the time of screening in the community, do you return to screen them? Yes/No
   1. If you do not return, what do you do to reach that individual?:_____________

***OPEN-ENDED***

1. What are some of the challenges in conducting screening in the community?
